# Serum Sorbitol Dehydrogenase as a Novel Prognostic Factor for Hepatocellular Carcinoma after Surgical Resection

**DOI:** 10.3390/cancers13236143

**Published:** 2021-12-06

**Authors:** Dongsub Jeon, Won-Mook Choi, Jin-Sun Kim, Yusun Jung, Su-Yeon Lee, Haeng Ran Seo, Kang Mo Kim

**Affiliations:** 1 Department of Gastroenterology, Asan Liver Center, Asan Medical Center, University of Ulsan College of Medicine, Seoul 05505, Korea; liver8665@amc.seoul.kr (D.J.); dr.choi85@amc.seoul.kr (W.-M.C.); jinsun1017@amc.seoul.kr (J.-S.K.); dandy7890@naver.com (Y.J.); 2 Advanced Biomedical Research Laboratory, Institut Pasteur Korea, Seongnam-si 13488, Korea; suyeon.lee@ip-korea.org

**Keywords:** hepatocellular carcinoma, recurrence-free survival, sorbitol dehydrogenase

## Abstract

**Simple Summary:**

A large percentage of patients with hepatocellular carcinoma (HCC) who undergo surgical resection experience a recurrence of their disease. Therefore, predicting recurrence after resection for HCC is crucial to select appropriate surgical candidates. The aim of this study was to determine if serum sorbitol dehydrogenase (SORD) levels, an enzyme that reflects liver damage, was associated with the length of recurrence-free survival. This study’s findings that serum SORD levels ≥15 ng/mL were associated with a shorter recurrence-free survival might help to determine which patients are better candidates for surgery in HCC. Moreover, baseline serum SORD and alpha-fetoprotein (AFP) levels could better predict the outcome when used in combination, with patients having both elevated SORD (≥15 ng/mL) and AFP (≥400 ng/mL) levels having a particularly poor prognosis. Therefore, incorporating serum SORD along with AFP levels in clinical practice may raise predictability of prognosis in HCC patients.

**Abstract:**

The majority of patients with hepatocellular carcinoma (HCC) undergoing curative resection experience tumor recurrence. To examine the association between preoperative serum sorbitol dehydrogenase (SORD), a liver-derived enzyme that reflects liver damage, and recurrence of HCC after curative resection, 92 patients were randomly selected who underwent curative resection for HCC between 2011 and 2012 from a prospective registry. Recurrence-free survival (RFS) was compared based on serum SORD levels. Cox proportional hazard models were used to investigate prognostic factors for RFS. During a median follow-up duration of 57.1 months, 43 patients experienced HCC recurrence. Patients with serum SORD ≥15 ng/mL (HR, 3.46; 95% CI, 1.76–6.81; *p* < 0.001) had worse RFS compared with patients with serum SORD <15 ng/mL. Serum AFP and SORD levels were two independent prognostic factors for RFS. When patients were stratified by baseline serum SORD and AFP levels, patients with serum AFP levels ≥400 ng/mL and serum SORD levels ≥15 ng/mL had a distinctly poor prognosis with the lowest RFS rates (HR, 22.08; 95% CI, 6.91–70.50; *p* < 0.001). Baseline serum SORD is an effective prognostic factor for HCC after resection. It may help guide patient selection for surgery, especially when combined with serum AFP levels.

## 1. Introduction

Hepatocellular carcinoma (HCC) is the most common type of primary liver cancer with a high prevalence and incidence in Asia [1]. Liver resection is the treatment of choice for patients with early-stage or resectable HCC [2], but the results are unsatisfactory. The survival rate remains low for these patients due to the high recurrence rate. The 5-year recurrence rate was 68% in patients with a single HCC (≤2 cm) after hepatectomy [3], and HCC recurrence adversely affects the long-term survival of patients [4,5]. Therefore, predicting recurrence after resection for HCC is crucial in order to select appropriate surgical candidates. Previous studies have noted that preoperative serum alpha-fetoprotein (AFP) levels and various histological features of tumors such as tumor size and microvascular invasion are independent predictors of recurrence after resection [6,7,8,9,10,11,12]. However, histological features are limited since they cannot be evaluated preoperatively. Moreover, AFP has a relatively low sensitivity and specificity for accurately predicting HCC [13], and the association of AFP with surgical outcome has been contradictory [14,15]. As a result, there is still a need for a novel prognostic marker to predict outcomes in patients with HCC after resection.

Inflammation, necrosis, and liver regeneration induced by various liver diseases play an important role in promoting HCC development [16]. More than 90% of HCCs develop in the context of hepatic damage and inflammation, making it a clear example of inflammation-related cancer. Sorbitol dehydrogenase (SORD), an enzyme in the polyol pathway converting sorbitol into fructose, reflects liver damage [17,18,19,20,21]. SORD is concentrated primarily in the liver similar to alanine aminotransferase (ALT) [22]. In patients with liver diseases including hepatitis, cirrhosis, and HCC, serum levels of SORD are elevated along with elevated levels of serum aspartate aminotransferase (AST) and ALT [23,24].

The polyol pathway, which produces sugar alcohols by aldo-keto reductase and SORD contributes to cancer development and aggressiveness [25,26]. In the previous study, blood sugar alcohol levels such as sorbitol increased steadily from healthy controls to patients with chronic liver disease and finally, HCC patients [27]. Moreover, increased expression of aldo-reductase and SORD was observed in various cancers such as liver, breast, and colorectal cancers [25,28]. A recent proteomics study found that the levels of SORD expression in tumor tissue were significantly associated with prognosis in patients with HCC [29,30], implying that serum SORD levels may be used as a prognostic marker in these patients. However, there has been no study evaluating the association between preoperative serum SORD levels and surgical outcomes of patients with HCC. This study aimed to evaluate the association between preoperative serum levels of SORD and HCC recurrence in patients with early-stage HCC after curative resection.

## 2. Results

### 2.1. Patient Baseline Characteristics

**Figure 1:**
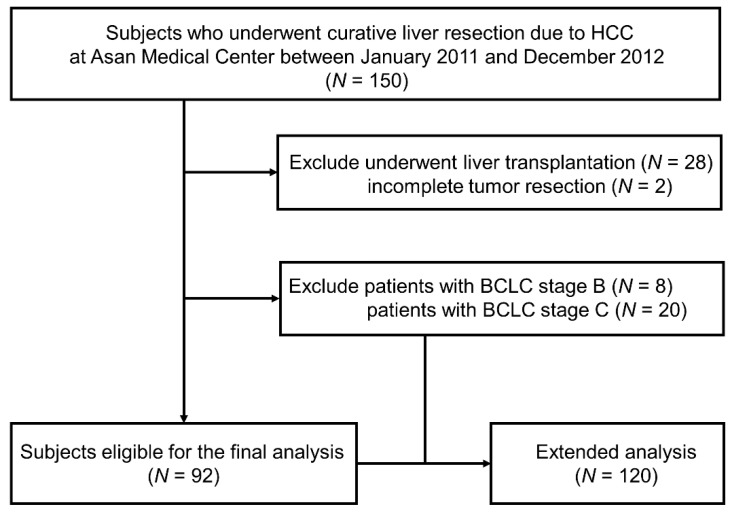
Patient flow chart.

In total, 92 patients who underwent curative-intent liver resection for HCC were included in the study (Figure 1). Their median age was 55.0 years, and most were male (76/92, 82.6%), had chronic hepatitis B as the etiology of HCC (82/92, 89.1%), had a Child-Pugh score of 5 (80/92, 87.0%), and received minor resection (74/92, 80.4%). Other demographics including liver function characteristics, treatment methods, and clinicopathologic factors are noted in Table 1. When the patients were divided into two groups according to preoperative baseline serum SORD level, 73 had a baseline SORD <15 ng/mL and 19 had a baseline SORD ≥15 ng/mL. All of the baseline characteristics were similar between the two groups except for resection type. Patients with baseline SORD ≥15 ng/mL had a major resection more frequently than those with a baseline SORD <15 ng/mL (Table 1).

### 2.2. Recurrence According to SORD Level

During the median follow-up time of 57.1 months, recurrence was observed in 43 patients. There was a significant difference in median serum SORD levels between those with and without recurrence, 10.0 ng/mL and 7.1 ng/mL, respectively. There were no significant differences in other baseline characteristics including hepatic functional reserve (Child-Pugh score, albumin-bilirubin (ALBI) grade, and indocyanine green (ICG) clearance) and tumor factors such as size, microscopic vascular invasion, and Edmondson grade between patients with and without recurrence (Supplementary Table S1).

**Figure 2:**
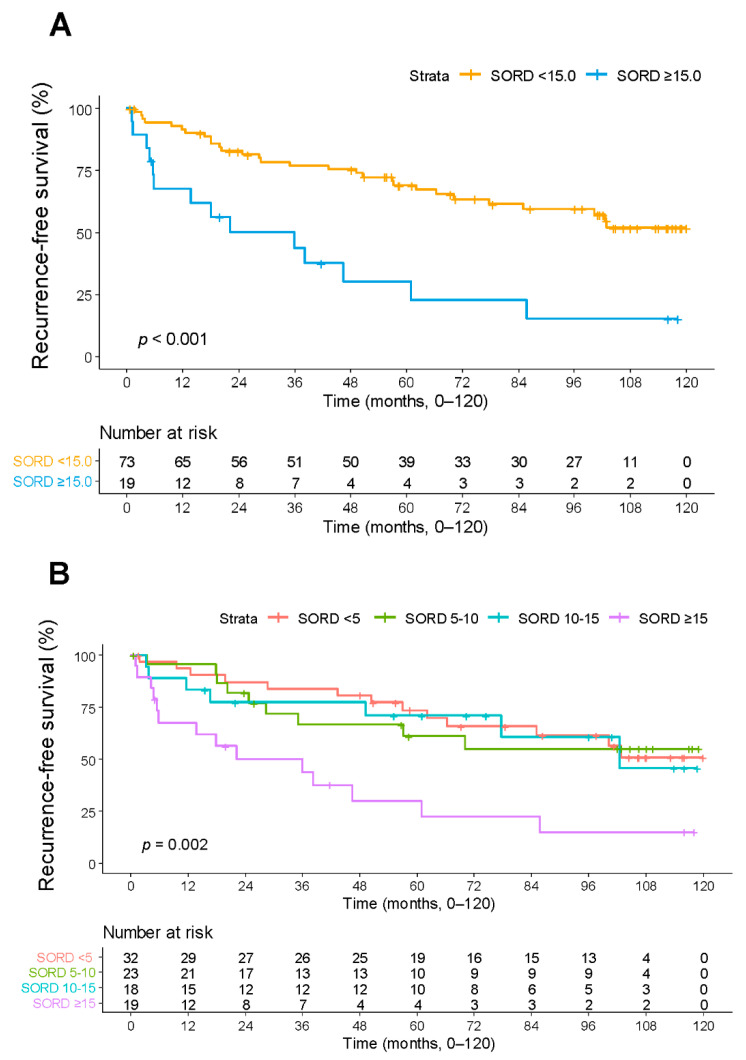
Kaplan–Meier plot for recurrence-free survival in patients with HCC after intent-to-cure resection stratified by serum SORD levels. (**A**) Patients stratified into two groups by baseline serum SORD level (<15 ng/mL and ≥15 ng/mL); (**B**) Patients stratified into four groups by baseline serum SORD level (<5 ng/mL, 5–10 ng/mL, 10–15 ng/mL, and ≥15 ng/mL).

Kaplan–Meier estimates of recurrence-free survival (RFS) are noted in Figure 2. In total, median and 2-year RFS rates following curative-intent resection of HCC were 100.3 months and 76.3%, respectively. When subjects were stratified by baseline serum SORD levels into two groups (≥15 ng/mL vs. <15 ng/mL), the group with high levels had a worse outcome with a 2-year RFS rate of 50.1% compared to a 2-year RFS rate of 83.0% (*p* < 0.001) for those with low serum SORD levels (Figure 2A). When the patients were classified into four groups according to baseline serum SORD levels (<5, 5–10, 10–15, ≥15 ng/mL), the RFS was similar among all patients with levels <15 ng/mL (Figure 2B). However, RFS was significantly lower in patients with SORD levels ≥15 ng/mL compared with all other groups.

When patients with Barcelona Liver Cancer (BCLC) stage B or C disease were included in the extended analysis, the results were similar to the primary analysis with worse outcomes in patients with baseline serum SORD levels ≥15 ng/mL (Supplementary Figure S1).

### 2.3. Prognostic Factors Associated with Recurrence-Free Survival

Univariate and multivariable Cox proportional hazard regression analyses were conducted to investigate the prognostic factors for RFS after curative-intent liver resection (Table 2).

In the multivariable regression analysis, high serum α-fetoprotein (AFP) levels (≥400 ng/mL; hazard ratio (HR), 2.08; 95% confidence interval (CI, 1.04–4.17; *p* = 0.04) and high serum SORD levels (≥15 ng/mL; HR, 3.24; 95% CI 1.64–6.37; *p* < 0.001) were identified as independent prognostic factors for RFS (Table 2 and Supplementary Figure S2).

**Figure 3:**
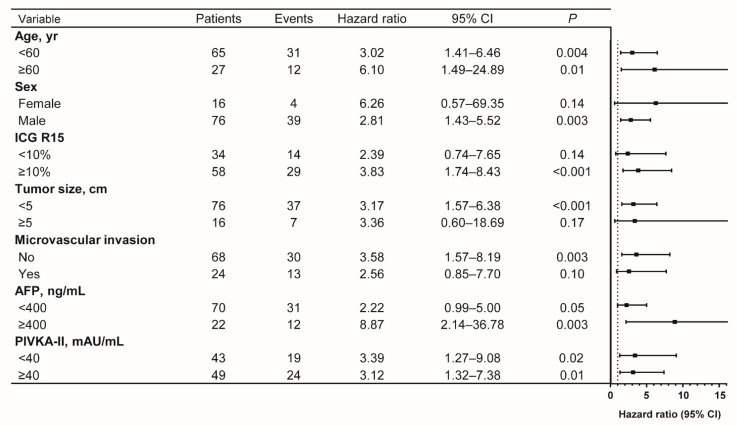
Forest plot of recurrence-free survival by baseline serum SORD levels in patient subgroups. Hazard ratio for patients with elevated serum SORD levels (≥15 ng/mL) compared with patients with low serum SORD levels (<15 ng/mL) as a reference.

When subgroup analyses were conducted, patients with high baseline SORD levels had worse RFS across all subgroups compared with low baseline SORD levels (Figure 3).

**Figure 4:**
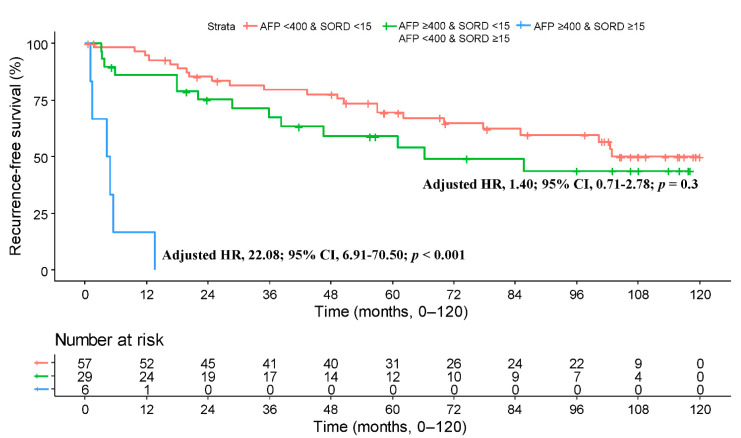
Kaplan–Meier plot for recurrence-free survival stratified by serum levels of AFP and SORD. Hazard ratio adjusted for sex and ICG R15.

Of note, the effect size of baseline SORD level on RFS was higher in patients with elevated serum AFP levels (≥400 ng/mL; HR, 8.87; 95% CI, 2.14–36.78; *p* = 0.003) compared with those with low serum AFP levels (<400 ng/mL; HR, 2.22; 95% CI, 0.99–5.00; *p* = 0.05). This suggests that baseline serum SORD levels are particularly predictive of outcome in patients with high serum AFP levels and could further stratify patients at risk along with serum AFP levels. Based on this result, when patients were stratified by baseline serum SORD and AFP, patients with both elevated AFP and SORD levels had a distinctly poor prognosis with the lowest RFS rates (HR, 22.08; 95% CI, 6.91–70.50; *p* < 0.001). The RFS rates of the other two groups was similar (HR, 1.40; 95% CI, 0.71–2.78; *p* = 0.30) (Figure 4).

### 2.4. Factors Correlated with Serum SORD

Spearman’s rank correlation coefficient was calculated to investigate factors correlated with SORD levels (Supplementary Figure S3). While SORD levels were positively correlated with indocyanine green retention rate at 15 min (ICG-R15) (r = 0.27; *p* = 0.008) and AST (r = 0.23; *p* = 0.03) and were negatively correlated with albumin (r = −0.26; *p* = 0.01), there was no correlation between SORD levels and other tumor markers including AFP (r = 0.002; *p* = 0.99) and protein induced by vitamin K absence or antagonist-II (PIVKA-II) (r = 0.08, *p* = 0.46).

Upon evaluating the correlation between pathological characteristics of HCC and baseline serum SORD levels, the presence of microvascular invasion or Edmonson-Steiner grade was not associated with baseline SORD levels (Supplementary Figure S4A). However, subjects with higher ALBI grades tended to have higher serum SORD levels (Supplementary Figure S4B). When patients with BCLC B or C were included in an extended analysis, patients with higher ALBI grades or a higher Child-Pugh score had higher baseline serum SORD levels (Supplementary Figure S4C), indicating that baseline serum SORD levels may reflect underlying hepatic functional reserve.

## 3. Discussion

This study evaluated the association between preoperative baseline serum SORD levels and surgical outcome in patients who underwent curative resection for HCC. Patients with high baseline serum SORD levels (≥15 ng/mL) had a significantly worse RFS of 50.1% at 2 years compared with a 2-year RFS of 83.0% for those with low baseline serum SORD levels (<15 ng/mL). Based on a multivariable Cox regression analysis, a high SORD level was a statistically significant prognostic factor for RFS after curative-intend resection for HCC. Of note, on subgroup analysis, baseline SORD levels were highly predictive of surgical outcome, especially in patients with an elevated serum AFP level (≥400 ng/mL) compared with those with low serum AFP levels (<400 ng/mL). When patients were stratified by baseline serum AFP and SORD levels, patients with both elevated baseline AFP and SORD levels had a distinctly poor prognosis.

SORD is an enzyme that is present in multiple tissues throughout the body but is primarily found in the liver [22,31]. Normally, serum SORD levels are low, but when liver damage is present, levels increase along with ALT and AST [32]. This suggests that elevated SORD levels are indicative of hepatocellular damage [19,33]. Chronic hepatocellular damage and hepatocyte necrosis leads to myofibroblast activation, resulting in liver fibrosis and cirrhosis. In a cirrhotic liver, continuous hepatocellular damage contributes to carcinogenesis by disrupting telomeres, releasing reactive oxygen species, and altering paracrine signaling in the cellular microenvironment [34,35]. Additionally, a previous proteomic study has noted that low SORD expression in liver tissue of patients with HCC is related to poor survival [29]. Intriguingly, another study found an inverse relationship between serum and liver SORD activity in a rat model with chemically induced liver injury [24]. Based on these findings, it may be assumed that patients with high serum SORD levels have low hepatic expression of SORD, which is indicative of poor survival outcomes in this group of patients. When SORD activity is low or absent in the liver, sorbitol can accumulate in hepatocytes during hyperglycemia [36]. The accumulation of sugar alcohols, including sorbitol, in the liver from a paucity of SORD activity may contribute to hepatocarcinogenesis [27]. Additionally, activation of glycolytic pathways such as the polyol pathway in cancer cells, of which SORD is a key enzyme, may contribute to further accumulation of sorbitol, promoting the poor differentiation of HCC [28]. Moreover, sorbitol by osmotic stress can activate the c-Jun N-terminal kinase, p38 mitogen-activated protein kinase, and mammalian target of rapamycin pathways, which may further promote the proliferation of HCC [37,38]. In this regard, a recent study demonstrated that the polyol pathway inhibitor enhances anti-cancer effects of sorafenib by blocking the mTOR pathway, suggesting that the activation of the polyol pathway in HCC may facilitate multityrosine kinase inhibitor escape via alternative pathways [39,40].

In the current study, elevated serum AFP levels (≥400 ng/mL) and elevated serum SORD level (≥15 ng/mL) were identified as two independent prognostic factors for RFS based on multivariable Cox regression analysis. An elevated AFP level is known to be associated with poor survival after hepatectomy [41]. Elevated serum AFP levels are associated with massive or infiltrative tumor type and portal vein tumor thrombus [42]. However, to date, there is still controversy over the role of AFP in predicting surgical outcomes in patients with HCC [14,15,43]. In the present study, the effect size of SORD for predicting surgical outcome was larger than that of AFP. Interestingly, interaction (subgroup) analyses revealed that SORD and AFP are interactive for predicting surgical outcome, and the predictive ability of SORD is enhanced in patients with an elevated AFP level. This result suggests that SORD may complement the prognostic ability of AFP in patients with HCC receiving curative-intent resection. Indeed, patients with both elevated SORD and AFP levels had a grave prognosis with <6 months median RFS.

Other well-known prognostic factors for HCC recurrence after curative-intent resection are microvascular invasion and tumor size [6,7,8,9]. However, in the current study, those histological factors were not significantly associated with RFS. This could be due to the baseline characteristics of the patients who were all BCLC stage 0 or A and had small tumors (median, 3.0 cm). Patients with advanced BCLC stage and large tumor size were not included in this study because curative-intent resection is not the standard of care for these patients. Presence of microvascular invasion is known to be one of the most important risk factors affecting post-operative tumor recurrence [8,44,45], which was not replicated in our study. This is mostly due to type 2 error because of the small number of patients included in our study. Nevertheless, when we further included presence of microvascular invasion in the multivariable analysis, the results that showed a poorer recurrence-free survival of patients with elevated serum SORD levels (≥15 ng/mL) remained unchanged.

To the best of our knowledge, there are no previous studies investigating the correlation between other prognostic factors for HCC and serum SORD levels. In this study, there was no significant association between SORD levels and tumor prognostic factors such as tumor size, AFP, and PIVKA-II. However, SORD levels were positively associated with AST levels, ICG-R15, and ALBI grades, which reflects liver damage or hepatic functional reserve. SORD levels have the advantage of predicting prognosis as well as reflecting hepatic functional reserve compared with other conventional tumor markers such as AFP and PIVKA-II. However, further studies are needed to confirm the ability of SORD levels to evaluate hepatic functional reserve.

Since it is easier to obtain blood samples as opposed to tissue samples, SORD may be a more efficient and useful prognostic predictor in clinical practice for a patient with HCC, even though further studies are needed to validate the current findings. Also, the study subjects were randomly selected from a prospective registry, minimizing any selection bias. However, there are some limitations to this study. First, matched tumor samples to measure SORD levels or activity were unavailable. Therefore, the inverse association between serum and tissue SORD levels could not be documented as postulated previously. Second, the number of patients included was small, and these results need further validation with a larger number of patients. Third, this research was conducted in a hepatitis B virus-endemic area, where the most common cause of HCC is hepatitis B virus infection. Thus, these results require further validation in HCC associated with other underlying liver diseases. Fourth, our study was a retrospective cohort study including randomly selected patients from prospective registry, which has intrinsic limitations such as bias and confounding. Further well-designed prospective study aiming to corroborate our results is needed.

## 4. Materials and Methods

### 4.1. Study Design and Population

From January 2011 to December 2012, the records of 150 patients who underwent curative liver resection due to HCC at Asan medical center (Seoul, Korea) and had peripheral blood stored at the Bio-Resource Center of the Asan Medical Center (BRC, Seoul, Korea) were randomly abstracted and included for analysis. The patients had all signed informed consent for the use of this information in future research. Exclusion criteria consisted of: (a) patients who underwent liver transplantation, (b) patients with incomplete tumor resection, and (c) patients who were classified as BCLC stage B or C. These BCLC stage B or C patients were later included in an extended analysis. Ultimately, 92 patients were included in the primary analysis and 120 patients in the extended analysis. The study protocol was approved by the Institutional Review Board of the Asan Medical Center (No. 2020-1173) which waived the requirement for informed consent due to the retrospective nature of the study. This study was performed in accordance with the Declaration of Helsinki.

### 4.2. SORD Measurement

SORD was measured by serum samples attained from the Bio-Resource Center of the Asan Medical Center. Serum samples were stored in a fresh-frozen state at −196 °C. After defrosting the samples, sufficient pre-incubation time of 24 h was used before initiating the enzyme reaction. This increased the accuracy of the measurements because metabolites in serum, especially ketones, can react with SORD in serum. The baseline serum levels of SORD were measured using Human Sorbitol dehydrogenase ELISA Kits (MyBioSource, San Diego, CA, USA) following the manufacturer’s instructions.

### 4.3. Risk Factors and Outcome

Clinicopathological data, including hepatic functional reserve assessment by Child-Pugh classification, ALBI grade, and ICG-R15 was collected. Liver resection was classified as major if ≥4 segments (according to the Couinaud classification) were resected and as minor resection if <4 segments were resected. Tumor size was defined as the diameter of the largest tumor in the surgical specimen.

The outcome of interest was RFS. RFS was defined as the time interval between the date of operation and the date of recurrence. Multiphasic computed tomography or magnetic resonance imaging was performed as well as measuring tumor markers, including serum AFP and PIVKA-II. 1 month after resection, then at 3-month intervals in the first 2 years, and every 3 to 6 months in subsequent years. All patients were followed from the date of operation to the date of tumor recurrence or death or until 31 December 2020.

### 4.4. Statistical Analysis

Descriptive statistics were presented as median (interquartile range and number with percentage for continuous and categorical variables, respectively. Continuous variables were compared using the Mann-Whitney U test. Categorical variables were compared using Fisher’s exact test or the chi-square test as appropriate. Survival curves for time-to-event outcomes were determined using the Kaplan–Meier analysis, and they were compared using a log-rank test according to baseline SORD levels. HRs for RFS and their 95% CI were calculated using a Cox proportional hazard model after checking the proportional hazards assumption of the variables. Spearman’s rank correlation coefficient was estimated between baseline SORD levels and other prognostic factors for RFS. Statistical analyses were performed using R statistical software, version 3.5.0 (R Foundation for Statistical Computing, Vienna, Austria; http://cran.r-project.org/; accessed on 1 May 2021). All tests were two-sided, with *p* < 0.05 considered statistically significant.

## 5. Conclusions

In conclusion, a baseline, elevated preoperative serum SORD level (≥15 ng/mL) was significantly associated with poor prognosis in patients with HCC after curative-intend resection. Moreover, baseline serum SORD and AFP levels could better predict the outcome, with patients having both elevated SORD (≥15 ng/mL) and AFP (≥400 ng/mL) levels having a particularly poor prognosis. Based on these findings, incorporating serum SORD along with AFP levels in clinical practice may assist with decision-making regarding appropriate surgical candidates and avoiding unnecessary surgery in patients with an expected poor survival.

## Figures and Tables

**Table 1:** Study population baseline characteristics.

Characteristics	Total	SORD <15 ng/mL	SORD ≥15 ng/mL	*p-*Value
	[N = 92]	[N = 73]	[N = 19]	
Age, median [IQR], *y*	55.0 [47.8, 60.3]	54.0 [46.0, 61.0]	57.0 [51.0, 59.5]	0.40
Male, *n* (%)	76 (82.6)	58 (79.5)	18 (94.7)	0.22
Etiology, *n* (%)				0.79
Hepatitis B	82 (89.1)	65 (89.0)	17 (89.5)	
Hepatitis C	3 (3.3)	2 (2.7)	1 (5.3)	
Others	7 (7.6)	6 (8.2)	1 (5.3)	
Diabetes, *n* (%)	19 (20.7)	12 (16.4)	7 (36.8)	0.10
Previous TACE, *n* (%)	15 (16.3)	11 (15.1)	4 (21.1)	0.78
AFP, ng/mL	32.1 [6.3, 300.5]	51.4 [6.4, 270.0]	11.6 [7.0, 1501.5]	0.84
<400, *n* (%)	70 (76.1)	57 (78.1)	14 (68.4)	0.56
≥400, *n* (%)	22 (23.9)	16 (21.9)	6 (31.6)	0.56
PIVKA-II, mAU/mL	45.0 [21.8, 366.5]	45.0 [23.0, 309.0]	68.0 [19.0, 495.5]	0.91
<40, *n* (%)	43 (46.7)	34 (46.6)	9 (47.4)	>0.99
≥40, *n* (%)	49 (53.3)	39 (53.4)	10 (52.6)	>0.99
AST, median [IQR], IU/L	31.5 [26.8, 44.0]	31.0 [26.0, 42.0]	39.0 [28.0, 56.0]	0.18
ALT, median [IQR], IU/L	33.0 [24.0, 44.5]	32.0 [24.0, 43.0]	38.0 [28.0, 53.0]	0.24
Platelet, median [IQR], ×10^3^/μL	131.50 [110.8, 175.5]	131.0 [113.0, 178.0]	132.0 [102.0, 159.5]	0.53
Albumin, median [IQR], g/dL	3.9 [3.6, 4.2]	3.9 [3.6, 4.2]	3.7 [3.5, 4.2]	0.32
Bilirubin, median [IQR], IU/L	0.9 [0.7, 1.1]	0.9 [0.7, 1.1]	0.9 [0.7, 1.2]	0.89
Creatinine, median [IQR], mg/dL	0.8 [0.7, 0.9]	0.8 [0.7, 0.9]	0.8 [0.8, 1.0]	0.64
Prothrombin time, median [IQR], INR	1.06 [1.01, 1.13]	1.06 [1.01, 1.13]	1.06 [1.01, 1.14]	0.60
Child-Pugh score, *n* (%)				0.67
5	80 (87.0)	63 (86.3)	17 (89.5)	
6	9 (9.8)	8 (11.0)	1 (5.3)	
7	3 (3.3)	2 (2.7)	1 (5.3)	
ALBI grade, *n* (%)				0.56
1	38 (41.3)	31 (42.5)	7 (36.8)	
2	52 (56.5)	41 (56.2)	11 (57.9)	
3	2 (2.2)	1 (1.4)	1 (5.3)	
ICG-R15, median [IQR], %	11.2 [8.2, 13.8]	11.0 [8.2, 13.4]	13.6 [8.9, 16.8]	0.23
Cirrhosis	81 (88.0)	64 (87.7)	17 (89.5)	>0.99
Resection type *, *n* (%)				0.01
Major	18 (19.6)	10 (13.7)	8 (42.1)	
Minor	74 (80.4)	63 (86.3)	11 (57.9)	
Tumor size, median [IQR], cm	3.0 [2.3, 4.2]	3.0 [2.1, 4.3]	3.3 [2.8, 3.5]	0.32
Microscopic vascular invasion, *n* (%)	24 (26.1)	16 (21.9)	8 (42.1)	0.14
Edmondson grade, *n* (%)				>0.99
I or II	19 (20.7)	15 (20.5)	4 (21.1)	
III or IV	73 (79.3)	58 (79.5)	15 (78.9)	
Recurrence, *n* (%)	43 (46.7)	29 (39.7)	14 (73.7)	0.02
Follow up, median [IQR], month	57.1 [19.8, 102.6]	66.3 [25.7, 104.3]	19.7 [5.3, 44.1]	0.003

Continuous variables with non-normal variables are reported as median (interquartile range [IQR]); and categorical variables are reported as number with percentage (%). * Major resection was defined as resection of four or more liver segments, with the remaining procedures considered as minor resection. AFP, α-fetoprotein; ALBI grade, albumin-bilirubin grade; AST, aspartate aminotransferase; ALT, alanine aminotransferase; ICG-R15, indocyanine green retention rate at 15 min; INR, international normalized ratio; IU, international unit; IQR, interquartile range; PIVKA-II, protein induced by vitamin K absence or antagonist-II; SORD, sorbitol dehydrogenase; TACE, transcatheter arterial chemoembolization.

**Table 2:** Univariate and multivariable analyses for recurrence-free survival.

Variable	Univariate Analysis			Multivariable Analysis		
	HR	95% CI	*p*-Value	HR	95% CI	*p*-Value
Age ≥60 *y*	1.02	0.52–1.98	0.96	—	—	—
Male	2.57	0.92–7.18	0.07	1.93	0.67–5.53	0.22
ICG R15 ≥10%	1.71	0.90–3.36	0.10	1.67	0.87–3.18	0.13
Cirrhosis	3.14	0.76–13.0	0.11	2.82	0.67–11.84	0.16
Child-Pugh score ≥6	0.77	0.30–1.97	0.59	—	—	—
ALBI grade ≥2	1.37	0.74–2.54	0.31	—	—	—
Tumor size ≥5 cm	0.98	0.44–2.21	0.97	—	—	—
Microscopic vascular invasion	1.48	0.77–2.83	0.24	—	—	—
AFP ≥400 ng/mL	1.68	0.86–3.27	0.13	2.08	1.04–4.17	0.04
PIVKA-II ≥40 mAU/mL	1.24	0.68–2.27	0.48	—	—	—
SORD ≥15 ng/mL	3.29	1.72–6.28	<0.001 *	3.24	1.64–6.37	<0.001
Major hepatectomy *	1.63	0.80–3.30	0.18	—	—	—

* Major resection was defined as resection of four or more liver segments. AFP, α-fetoprotein; ALBI grade, albumin-bilirubin grade; CI, confidence interval; HR, hazard ratio; ICG-R15, indocyanine green retention rate at 15 min; PIVKA-II, protein induced by vitamin K absence or antagonist-II; SORD, sorbitol dehydrogenase.

## Data Availability

The data presented in this study are available on request from the corresponding author. The data are not publicly available due to the permission issue of participants.

## Supplementary Materials

The following are available online at https://www.mdpi.com/article/10.3390/cancers13236143/s1, Figure S1: Kaplan–Meier plot for recurrence-free survival including BCLC stage B and C, Figure S2: Forest plot of multivariable analysis for recurrence-free survival, Figure S3: Heatmap of Spearman’s correlation coefficients among variables, Figure S4: Baseline serum SORD levels stratified by tumor histology and underlying liver function, Table S1: Baseline characteristics stratified by postoperative tumor recurrence.

## Abbreviations

AFP: α-fetoprotein; ALBI grade, albumin-bilirubin grade; AST, aspartate aminotransferase; ALT, alanine aminotransferase; BCLC, Barcelona Clinic Liver Cancer; BRC, Bio-Resource Center of the Asan Medical Center; CI, confidence interval; CT, computed tomography; HCC, hepatocellular carcinoma; HR, hazard ratio; ICG-R15, indocyanine green retention rate at 15 min; INR, international normalized ratio; IU, international unit; IQR, interquartile range; MRI, magnetic resonance imaging; PIVKA-II, protein induced by vitamin K absence or antagonist-II; RFS, recurrence-free survival; SORD, sorbitol dehydrogenase; TACE, transcatheter arterial chemoembolization.
