# Supplementary material for: Serum Sorbitol Dehydrogenase as a Novel Prognostic Factor for Hepatocellular Carcinoma after Surgical Resection
Source: Cancers (Basel). 2021 Dec 6;13(23):6143. doi: 10.3390/cancers13236143 (PMC8657083; doi:10.3390/cancers13236143)
Supplement: Supplementary file 1 [file cancers-13-06143-s001.zip › cancers-1453915-supplementary.pdf]

Supplementary Material: Serum Sorbitol Dehydrogenase as a Novel Prognostic Factor for Hepatocellular Carcinoma after Surgical Resection

Dongsub Jeon, Won-Mook Choi, Jin-Sun Kim, Yusun Jung, Su-Yeon Lee, Haeng Ran Seo and Kang Mo Kim

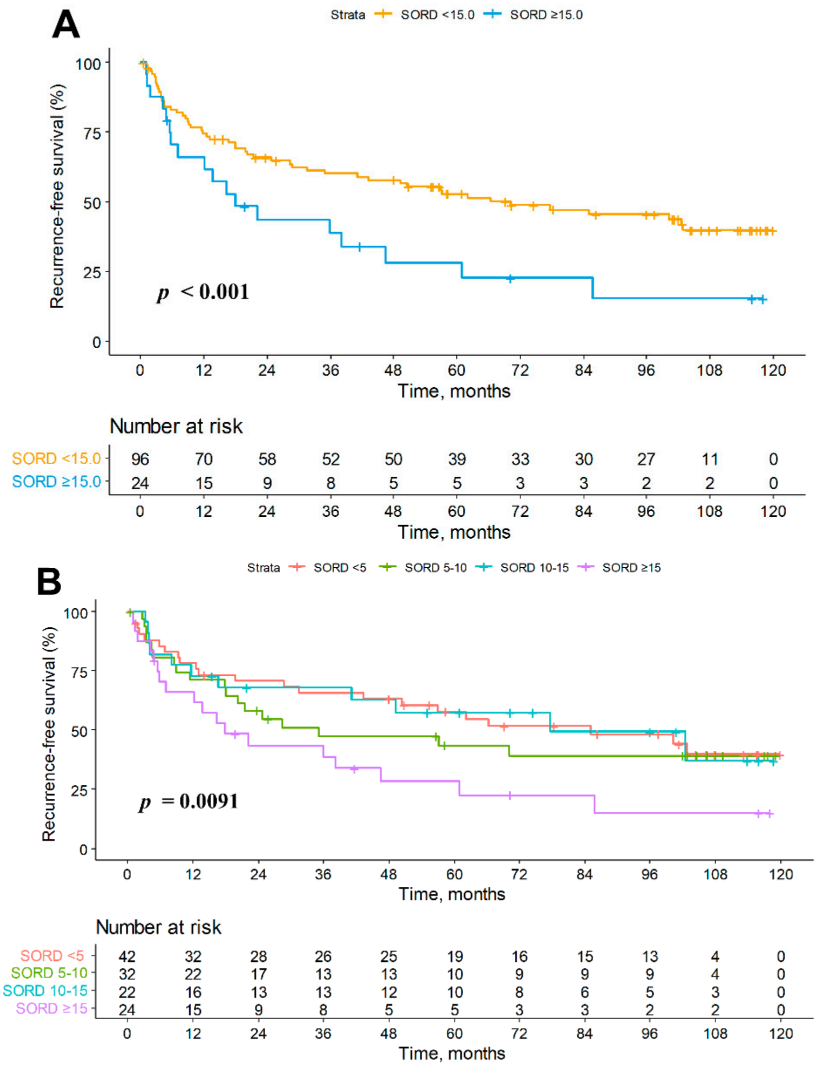

Figure S1. Kaplan–Meier plot for recurrence-free survival including BCLC stage B and C. (**A**) Patients stratified into two groups by baseline serum SORD level (<15 ng/mL and ≥15 ng/mL). (**B**) Patients stratified into four groups by baseline serum SORD level (<5 ng/mL, 5–10 ng/mL, 10–15 ng/mL, and ≥15 ng/mL).

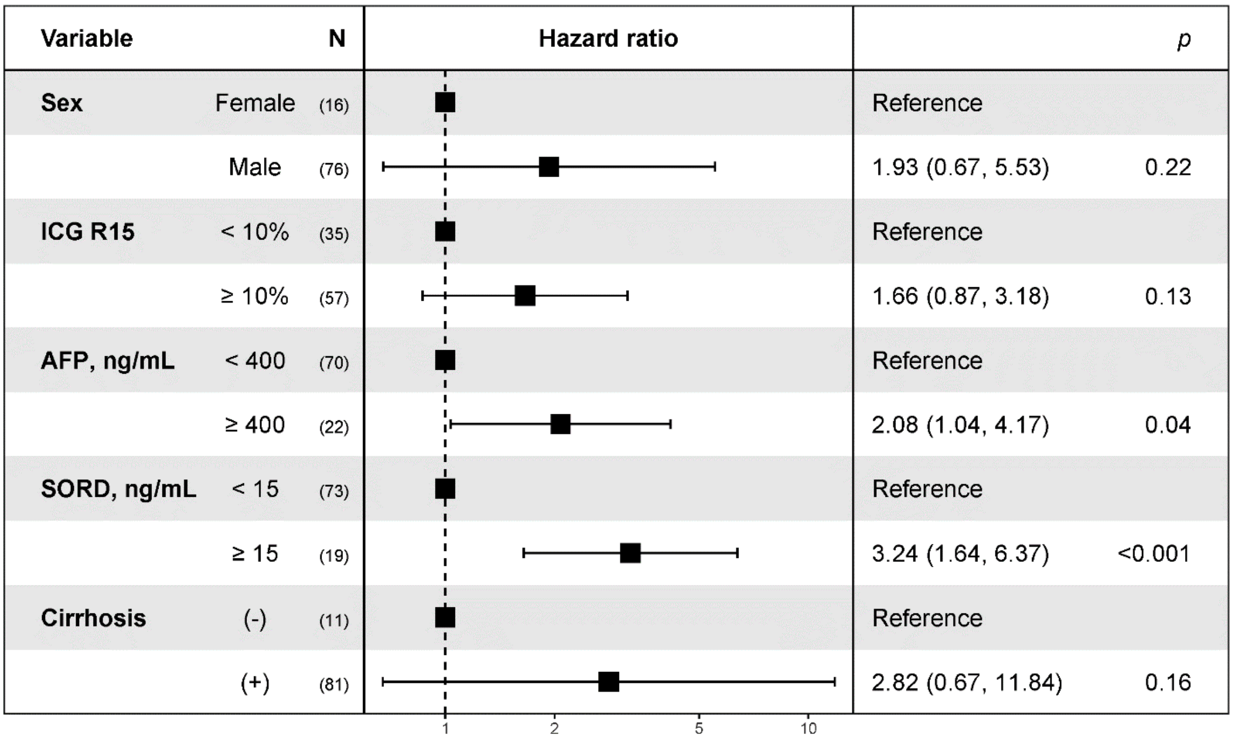

Figure S2. Forest plot of multivariable analysis for recurrence-free survival.

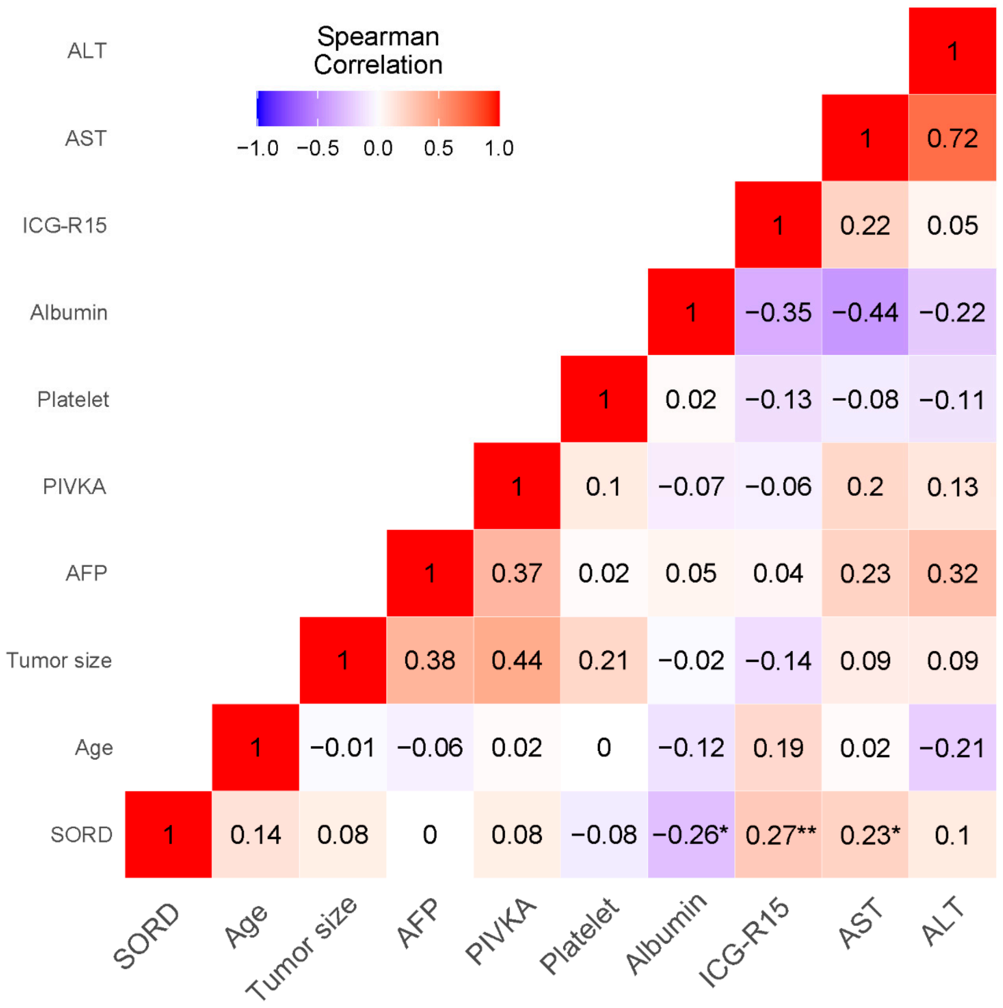

Figure S3. Heatmap of Spearman's correlation coefficients among variables. (*, ** indicate $p < 0.05$ and $p < 0.01$, respectively)

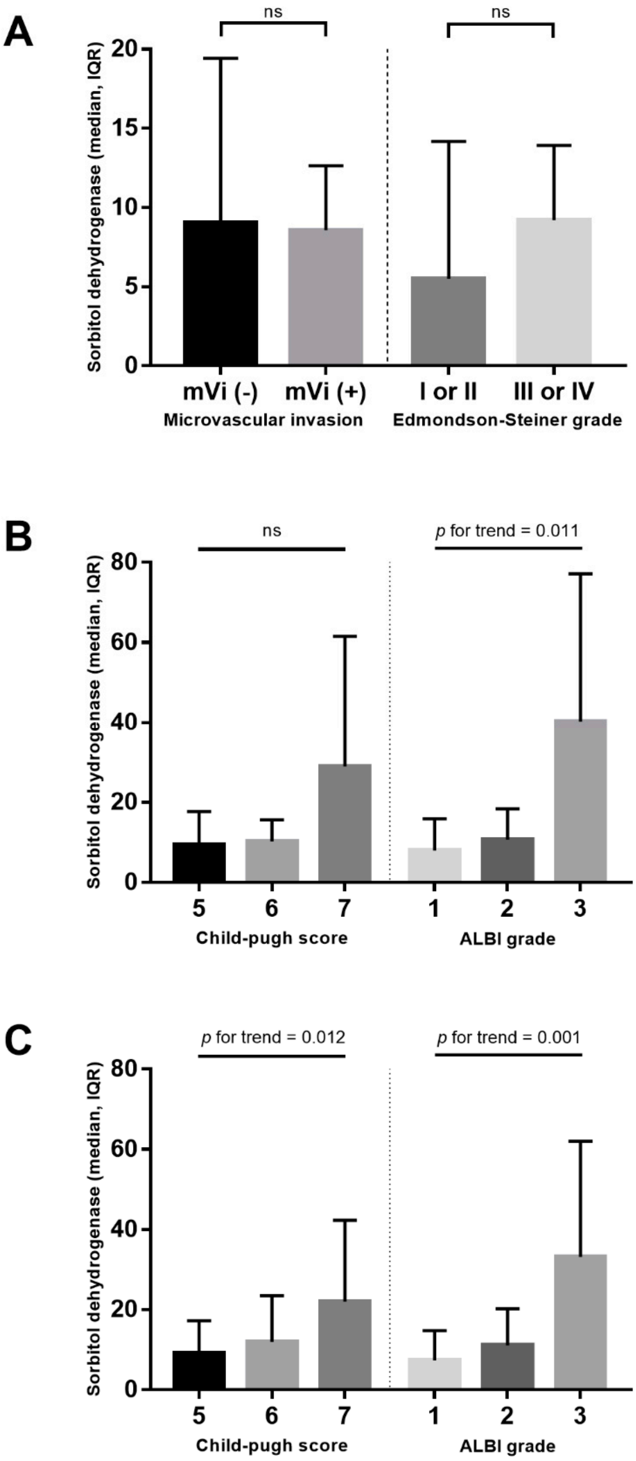

Figure S4. Baseline serum SORD levels stratified by tumor histology and underlying liver function. **(A)** Serum SORD levels stratified by the presence of microvascular invasion or Edmondson-Steiner grade (I or II vs. III or IV). **(B)** Serum SORD levels stratified by Child-Pugh score and ALBI grade in the primary analysis of subjects with BCLC 0 or A stage. **(C)** Serum SORD levels stratified by Child-Pugh score and ALBI grade in an extended analysis including subjects with all BCLC stages. (ns indicate $p > 0.05$)

Table S1. Baseline characteristics stratified by postoperative tumor recurrence.

Characteristics	No Recurrence [N = 49]	Recurrence [N = 43]	*P-Value*
Age, median [IQR], *y*	56.0 [48.0, 61.0]	54.0 [48.5, 60.0]	0.69
Male, *n* (%)	37 (75.5)	39 (90.7)	0.10
Etiology, *n* (%)			0.53
Hepatitis B	42 (85.7)	40 (93.0)	
Hepatitis C	2 (4.1)	1 (2.3)	
Others	5 (10.2)	2 (4.7)	
Diabetes, *n* (%)	11 (22.4)	8 (18.6)	0.84
Previous TACE, *n* (%)	5 (10.2)	10 (23.3)	0.16
SORD, median [IQR], ng/mL	7.1 [3.6, 11.6]	10.0 [4.6, 16.9]	0.040
AFP, ng/mL	22.8 [4.4, 253.0]	51.4 [9.3, 1009.1]	0.16
<400, *n* (%)	39 (79.6)	31 (72.1)	0.55
≥400, *n* (%)	10 (20.4)	12 (27.9)	0.55
PIVKA-II, mAU/mL	41.0 [22.0, 148.0]	51.0 [21.0, 632.5]	0.49
<40, *n* (%)	24 (49.0)	19 (44.2)	0.80
≥40, *n* (%)	25 (51.0)	24 (55.8)	0.80
AST, median [IQR], IU/L	29.0 [26.0, 40.0]	35.0 [28.0, 53.0]	0.09
ALT, median [IQR], IU/L	29.0 [22.0, 44.0]	35.0 [27.5, 45.5]	0.19
Platelet, median [IQR], x10^3/μL	131.0 [113.0, 166.0]	134.0 [107.5, 176.0]	0.81
Albumin, median [IQR], g/dL	3.9 [3.6, 4.2]	3.8 [3.6, 4.2]	0.96
Bilirubin, median [IQR], IU/L	0.8 [0.7, 1.0]	0.9 [0.7, 1.2]	0.74
Creatinine, median [IQR], mg/dL	0.8 [0.7, 0.9]	0.8 [0.7, 0.9]	0.56
Prothrombin time, median [IQR], INR	1.06 [1.01, 1.11]	1.06 [1.01, 1.13]	0.96
Child-Pugh score, *n* (%)			0.88
5	42 (85.7)	38 (88.4)	
6	5 (10.2)	4 (9.3)	
7	2 (4.1)	1 (2.3)	
ALBI grade, *n* (%)			0.95
1	21 (42.9)	17 (39.5)	
2	27 (55.1)	25 (58.1)	
3	1 (2.0)	1 (2.3)	
ICG-R15, median [IQR], %	11.0 [7.5, 13.1]	11.6 [8.8, 17.5]	0.10
Resection type*, *n* (%)			0.18
Major	8 (16.3)	10 (23.3)	
Minor	41 (83.7)	33 (76.7)	
Tumor size, median [IQR], cm	3.0 [2.2, 4.2]	3.0 [2.4, 4.2]	0.95
Microscopic vascular invasion, *n* (%)	11 (22.4)	13 (30.2)	0.54
Edmondson grade, *n* (%)			>0.99
I or II	10 (20.4)	9 (20.9)	
III or IV	39 (79.6)	34 (79.1)	

Continuous variables with non-normal distribution are reported as median (interquartile range [IQR]), and categorical variables are reported as number with percentage (%). * Major resection was defined as resection of four or more liver segments, with the remaining procedures considered to be minor resection. AFP, α-fetoprotein; ALBI grade, albumin-bilirubin grade; AST, aspartate aminotransferase; ALT, alanine aminotransferase; ICG-R15, indocyanine green retention rate at 15 minutes; INR, international normalized ratio; IU, international unit; IQR, interquartile range; PIVKA-II, protein induced by vitamin K absence or antagonist-II; SORD, sorbitol dehydrogenase; TACE, transcatheter arterial chemoembolization.
